# Supplementary material for: Synchronous diversification of Sulawesi's iconic artiodactyls driven by recent geological events
Source: Proc Biol Sci. 2018 Apr 11;285(1876):20172566. doi: 10.1098/rspb.2017.2566 (PMC5904307; doi:10.1098/rspb.2017.2566)
Supplement: Materials and Methods, Supplementary Tables and Supplementary Figures [file rspb20172566supp1.pdf]

Electronic Supplementary Materials

Materials and Methods:

Sampling

We obtained DNA or morphometric samples (traditional or geometric morphometric measurements), or both, from 456 Sulawesi warty pigs (SWP; *Sus celebensis*), 520 Anoa (*Bubalus* spp.), and 313 Babirusa (*Babyrousa* spp.). Sampling on Sulawesi can be difficult due to its remoteness and to recent population declines of endemic mammals. To overcome this limitation, we targeted the extensive collections of these three species in museums, private collections, local markets, and zoos across the world. All information necessary to assess the provenance, type of specimen, and more are provided as supplementary data (Table S1).

Taxonomic notes

We sampled individuals from the geographic locations (Togian, mainland Sulawesi, and Buru/Sula) of all three Babirusa species (Table S1). For Anoa, while the majority of our samples are from specimens with no species designation (*e.g.* museum samples collected prior to the split of Anoa into two species [1]) , our data set includes individuals assigned to both lowland and highland Anoa (Table S1). Given that the goal of this study is to understand the general evolutionary history of the island (and the fact that both Anoa and Babirusa are only found in Sulawesi and the neighboring islands), we treated all the Babirusa and Anoa samples as a single taxonomic unit. The relevance of the data presented here to our understanding of species designations will be addressed in future studies.

Morphometrics

A total of 356 teeth from 227 specimens (Babirusa: 76 M2 and 89 M3; SWP: 99 M2 and 92 M3) were measured and analysed using geometric morphometric approaches in 2D. We

strictly followed protocols developed by [2,3]. Differences in shape were tested using MANOVA, whereas differences in log-transformed centroid size were tested using Wilcoxon tests and visualized using boxplots. Variation in shape was first visualized using a principal-components analysis (PCA) before between-groups variation was explored using Canonical Variate Analyses (CVA). The resemblance between groups was visualized with a neighbor-joining network calculated on the Mahalanobis distances. Manova and CVA were performed after a dimensionality reduction of the data following [3]. The variances of the two species on Sulawesi were compared using a Fligner-Killeen test based on the distance between each specimen and the mean shape (or size) of its species. M2 and M3 were analysed separately before being pooled together to produce the synthetic Figure 2a.

Genetics

DNA extraction

We extracted DNA from 520 Anoa, 251 Babrirusa, and 317 SWPs. We sequenced mitochondrial cytochrome *b* (cytb) and D-loop (total length 1,394 bp) from 142 samples of Anoa, as well as partial D-loop from 213 and 230 samples of Babirusa (481 bp) and SWP (660 bp), respectively. We also typed 13 microsatellite loci for 163 samples of Anoa, 14 loci for 238 samples of SWP, and 13 loci for 182 samples of Babirusa. Genomic DNA was extracted from museum specimens, hair follicles and faeces using the DNeasy Blood and Tissue kit (Qiagen). DNA was quantified in a Nanodrop and visualized under UV light in 40 mL 1X TAE 1% agarose gels stained with SYBRsafe (Invitrogen).

For DNA extraction from bone, we grounded samples of cortical bone to powder in a Mikrodismembrator (Sartorius). We then digested bone powder overnight at 50 °C in 2 mL of buffer (0.425 M EDTA pH8, 1 mM Tris–HCl pH8, 0.05% w/v SDS, 0.33 mg/mL

Proteinase K) under constant rotation. The digested solution was concentrated to approximately 500 μL using 30 kDa molecular weight cut-off centrifugal filters (Amicon® Ultra, Millipore). We passed the concentrated solution through a silica column (QIAquick®, Qiagen) following the manufacturer's protocol, and eluted the final extract in 100 μL of TE buffer. We measured DNA concentration using 2 μL of extract on the Qubit® platform (Invitrogen), and stored the extracts at -20 °C.

mtDNA sequence data

From our samples of Anoa, we amplified D-loop and cytb fragments by polymerase chain reaction (PCR) using the primers described in Table S6. Both primers were designed by Dr D. Bradley (Trinity College, Dublin) to amplify the mtDNA of multiple bovine species [4,5]. Numerous samples were not sequenced due to the low quality of their DNA. Fragments were amplified by PCR using one cycle of denaturation at 96 °C for 3 min, followed by 30 cycles of: denaturation at 96 °C for 30 s, annealing at 50 °C for 20 s, and extension at 60 °C for 4 min. Both primers were run separately with an M13 tail added to the 5'-end. Sequencing was carried out using M13 universal primers and the ABI BigDye 3.1 sequencing kit (Applied Biosystems). Sequences were determined using an ABI 3700 automatic DNA capillary sequencer (Applied Biosystems), OrbixWeb™ Deamon software, 3700 DATA collection software and DATA Extractor software.

From our samples of Babirusa and SWP, we amplified two overlapping d-loop fragments for both species, which were amplified by PCR using primers designed by G. Larson (University of Oxford, UK)[6,7] and described in Table S6. PCR mixture was as follows: 2.5 μL x 5 Taq advanced buffer (containing 1.5 mM $MgCl_2$), 2.5 μL of each primer (10 μM), 0.5 μL 200 μM dNTPs, 0.25 μL 5 Prime Taq polymerase, 1 μL DNA (50–100 ng) adjusted to a final volume of 25 μL with ddH_2O. Fragments were amplified using one cycle of denaturation at 94 °C for 1 min 30 s followed by 40 cycles of: denaturation at 94 °C for 45

s, annealing at 53 °C for 45 s, extension at 72 °C for 1 min 30 s, followed by a final extension at 72 °C for 10 min. Each fragment of either marker was subjected to bidirectional sequencing using the ABI BigDye 3.1 sequencing kit (Applied Biosystems). Sequences were generated using an ABI 3130 DNA capillary sequencer (Applied Biosystems).

Microsatellite data

Anoa samples were genotyped for 13 bovine microsatellite loci using primers previously designed for cattle *Bos taurus* (with the forward primer fluorescently labelled): BM1818, CSRM60, ETH152, HAUT24, HAUT27, HEL13, ILSTS5, INRA35, INRA37, MM12, SPS115, TGLA126, and TGLA227. These loci were recommended by the Food and Agriculture Organization [8] for use in genetic diversity studies and were selected at the Roslin Institute (Edinburgh, UK). More details and the primer sequences are available in Table S6.

For some samples, PCR were done as simplex reactions in 10 µL final volume containing 1 µL 10X PCR buffer, 0.3 µL of 50 µM $MgCl_2$, 1 µL of each primer (10 µM), 1 µL of dNTPs (10 µM), 0.1 µL Platinum Taq polymerase, 4.6 µL of ddH_2O and 1 µL DNA (50–100 ng). Simplex PCR conditions were: initial denaturation at 94 °C for 3 min, followed by 30 cycles of: denaturation at 94 °C, annealing at 55–65 °C (depending on the marker) for 45 s and extension at 72 °C for 45 s, with a final extension of 72 °C for 3 min. For other samples, PCRs were done as multiplex reactions by pooling six or seven microsatellite primer pairs using the Type-It Microsatellite kit (Qiagen). Multiplex reactions were done in a final volume of 10 µL containing 5 µL 2X Type-It Master Mix, 1 µL 10X primer mix, 1 µL Q-solution, 1 µL ddH_2O and 2 µL DNA (50–100 ng). Multiplex PCR conditions followed the manufacturer's instructions (Qiagen). DNA from *Bos taurus* was used as a positive control.

Negative controls (without DNA) were included in all reactions. The PCR products were analysed using an ABI 373 (Applied Biosystems) DNA fragment analyser. Results were scored with the programs GENESCAN 3.0, GENOTYPER 2.5 or PEAK SCANNER 2.0 (Life Technologies).

For samples of SWP, PCR was performed in an Eppendorf Mastercycler® gradient apparatus. In general, the PCR profile was as follows: the 10 µL reaction mixture consisted of 1 µL DNA (about 50–100 ng), 1 x 5 Prime Taq advanced buffer (containing 1.5 mM $MgCl_2$), 1 µL of M13F (1 µM), 1 µL of each primer (10 µM) (0.5 µL for S0214 and S0149), 0.2 µL 200 µM dNTPs, 0.05 µL 5 Prime Taq DNA polymerase (0.1 µL for S0214 and S0149), 1 µL DNA (50–100 ng) adjusted to a final volume of 10 µL with ddH_2O. The thermal cycling, preceded by 5 min at 94 °C and followed by 5 min at 72 °C, consisted of 30 cycles (32 for S0386 and 35 for S0026) of 94 °C for 1 min, an optimal annealing temperature for 1 min (Table S6), and 72 °C for 1 min. PCR products were visualized on a 1.5% agarose gel (Acros organics) with GelRed Nucleic Acid Gel Stain (Biotium) in order to check the amplification.

Fragment analysis was performed on an ABI 310 (Life Technologies). For all markers, we used the M13 method to visualize the PCR products. To do so we added a M13 Forward (M13F; 5’-CACGACGTTGTAAAACGAC-3’) tag to the 5’ end of each forward primer. PCR mix contained 0.1 µM of this tag-labelled primer and 1 µM of both reverse primer and M13F labelled primer (0.05µM of tag-labelled primer and 0.5µM of reverse primer; and M13F labelled primer for markers S0149 and S0214). Data were interpreted and allele sizes determined using GeneMapper 4.0 software (Life Technologies).

For samples from Babirusa, PCRs were performed in an Eppendorf Mastercycler® gradient apparatus. In general, the PCR profile was as follows: the 10 µL reaction mixture consisted of 1 µL DNA (about 50–100 ng), 1 x Eppendorf Taq buffer containing 1.5 mM $Mg(Oac)_2$, 1 µM of each primer (0.5 µM for S0214 and S0149), 200 µM dNTPs (Eppendorf) and 0.25 U Taq DNA polymerase (0.5 U for S0214 and S0149). The thermal cycling, preceded by 5 min at 94 °C and followed by 5 min at 72 °C, consisted of 30 cycles (32 for S0386 and 35 for S0026) of 94 °C for 1 min, an optimal annealing temperature for 1 min (see Table S6), and 72 °C for 1 min. PCR products were visualized on a 1.5% agarose gel (Acros organics) with ethidium bromide (Merck) in order to check the amplification.

Fragment analysis was performed on an A.L.F. express DNA Sequencer (Pharmacia Biotech). For markers S0149 and S0228, we used the M13 method to visualize the PCR products. Hence, an M13 Forward (5’-CACGACGTTGTAAAACGAC-3’) tag was added to the 5’ end of each forward primer and the PCR mix contained 0.1 µM of this tag-labelled primer and 1 µM of the reverse primer as well as of the M13F-cy5 labelled primer (or 0.05 µM of the tag-labelled primer and 0.5 µM of the reverse and M13F-cy5 labelled primer in case of marker S0149). Data were interpreted and allele sizes determined using Genetools from SynGene and Allelelocator 1.03 software (Pharmacia Biotech). All primers are available in Table S6.

Phylogenetic analyses of mitochondrial DNA

A phylogenetic tree was inferred for each species, using MrBayes v3.2.5 [9](Figure S4; Figure S5; Figure S6). To estimate the position of the root, we included a sequence from *Phacochoerus africanus* (accession: AJ314533) for the analysis of Babirusa and SWP, and from *Bos taurus* (accession: EU177842) for the analysis of Anoa. The HKY+G

substitution model was selected, for each data-set, based on Bayes factors (marginal likelihood computed via stepping-stone sampling) of JC, HKY+G and GTR+G, with and without invariable sites. To estimate the posterior distribution of various parameters, we used Markov chain Monte Carlo sampling with 4 chains (comprising 3 heated chains and 1 cold chain) of 10,000,000 steps each (with samples drawn every 1000 steps). The first 25% of samples were discarded as burn-in. We carried out 4 independent MCMC analyses and combined the samples from the posterior. Convergence was assessed by ensuring that average standard deviation of split frequencies was below 0.01 and that the potential scale reduction factor was close to 1 for all parameters.

For each species we defined haplogroups based on highly supported clades. For each geographic region, the proportion of each haplogroup was plotted on a map using the R package "maps". For each sample, haplogroup membership was transposed to create an ancestry matrix. All samples lacking precise geographic coordinates were removed. The ancestry matrix was then plotted onto a map with a tessellated projection, using the R package "tess3r" [10–12]. We then divided Sulawesi and nearby islands into 11 regions based on previous work on amphibians and primates that defined areas of endemism on the island [13–15]. We assessed the significance of the difference in haplogroup frequency in each area of endemism using Pearson's chi-squared test, *p*-values were computed using 2000 simulation replicates, as implemented in R.

To infer the evolutionary timescales of the three species, we performed a Bayesian phylogenetic analysis using a molecular clock in BEAST v1.8.4 [16]. First, we analysed a mtDNA combined data set comprising the sequences of Babirusa, SWP and relatives (*S. cebifrons*, *S. philippensis*, *Hylochoerus meinertzhageni*, *Potamochoerus porcus*, *Potamochoerus larvatus*, *Phacochoerus aethiopicus*, and *Phacochoerus africanus*). This

data set comprised 700 aligned nucleotides from 243 samples. To calibrate the molecular clock, we used a normal calibration prior for the age of African suids (mean 10.5 My, standard deviation 2.551 My), based on the estimate from a combined nuclear and mitochondrial data set by [17].

We then analysed the mtDNA sequences of Anoa and related bovids (*Bison bison*, *Bison bonasus*, *Syncerus caffer*, *Bos taurus*, *Bos gaurus*, *Bos frontalis*, and *Bos grunniens*). This data set comprised 726 aligned nucleotides from 170 samples. We used a normal calibration prior for the age of the root (mean 8.8 My, standard deviation 1.02041 My), based on a fossil calibration used by [18]. Given the use of relatively deep calibrations in both analyses, the date estimates should be regarded as being conservatively old because our approach is likely to produce underestimates of the substitution rates [19].

The Bayesian information criterion was used to select the HKY+G model as the best-fitting substitution model for both data sets, after excluding models allowing a proportion of invariable sites. For each data set we compared two models of rate variation: the strict clock and the uncorrelated lognormal relaxed clock [20]. We also compared three tree priors: constant-size coalescent prior, Bayesian skyline coalescent prior, and birth-death speciation prior. For each combination of clock model and tree prior, the marginal likelihood was estimated using path sampling with 25 power posteriors [21]. Samples were drawn every 2,000 steps from a total of 2,000,000 MCMC steps per power posterior.

Posterior distributions of all parameters, including the tree, were estimated by MCMC sampling, with samples drawn every 5000 steps over a total of 50,000,000 MCMC steps. To ensure convergence, each analysis was run in duplicate and the samples were compared and combined. Sufficient sampling was confirmed by examining the effective

sample sizes of parameters. For both data sets, the strict clock and Bayesian skyline tree prior yielded the highest marginal likelihood (Table S7).

Analyses of microsatellite data

For each species, we used STRUCTURE v2.3.4 [22] to infer population structuring. The maximum number of populations (*K*) was set to 12 (the total number of region defined on Sulawesi). For each species, we ran 10 independent MCMC analyses, each with 1,000,000 steps, discarding a burn-in of 50,000 steps. We computed ΔK (Figure S8) to infer the best-fitting *K* value using structure Harvester [23]. Independent runs were merged using *CLUMPP* with *M*=2 [24]. For all samples with precise geographic coordinates, results were plotted onto a map with a tessellated projection, using the R package “tess3r” [10–12]. Results were also plotted on a map using the R package “maps” in each region of endemism (see above). To limit the possibility of provenance uncertainty, we excluded all samples that were from zoos or from unknown locations from this analysis (see Table S1).

We used the package hierfstat v0.04 [25] in R to compute Weir and Cockerham’s *Fst* [26]. Analyses of molecular variance (AMOVA)[27] were also performed in R using the package poppr v2.3.0 [28] and ade4 v1.7 [29] using populations as defined in Figure 4. We built neighbour-joining trees based on pairwise proportions of shared alleles [30](POSA; Figure S12) using PHYLIP [31]. For Babirusa and SWP we also computed average square distance (ASD) [32] between every pair of samples at 13 microsatellite loci (shared between SWP and Babirusa) in order to estimate the relative TMRCAs of these species [33]. Both ASD and POSA were computed using Microsatellite Analyser v3.13[34].

Geographical origins of population expansions

To infer the location of origin of population expansion for each species, we employed a spatially explicit discriminative modelling approach in which we assume a monotonic decline in diversity with distance from origin of a range expansion. A spatial grid of latitude and longitude values covering the geographic space of Sulawesi, of resolution 0.05 by 0.05 degrees, was explored using a flat kernel of radius 500 km for SWP and Babirusa and 350 km for Anoa*.* If at any location in the grid we found within the kernel at least 5 sampled individuals for SWP, or 3 sampled individuals for Babirusa and Anoa, the local diversity was calculated using ASD and recorded for that grid location. The grid was then re-explored with each latitude/longitude location treated as a potential origin location, and we recorded the correlation between geographic distance to the accepted kernels and local diversity at those kernels. This provided a grid of correlation values, which was then interpolated and visualized on a map.

Regions with the highest negative correlations were considered the best hypothesized origin locations. To quantify statistical support for inferred origin locations, the data were permuted among sample sites 1000 times, and for each permuted data set the above analysis was repeated. Following this, we plotted only the grid locations where the negative correlation between geographic distance and genetic diversity was more extreme than 99% (98% for Anoa) of those obtained from the permuted data.

Approximate Bayesian computation

For each species, we used both mtDNA and microsatellite data to evaluate the fit of four different models (Figure S11) and to obtain a posterior distribution of the parameters under the best-fitting model. We compared the fit of models with constant population size (Figure S11a), population expansion (Figure S11b), a bottleneck (Figure 10c), and a bottleneck following an expansion (Figure 10d). The rationale behind these models is to test whether

these species have undergone a population expansion due to the uplift of Sulawesi (see main text) and/or if they have undergone a bottleneck due to recent human activities. The prior distributions used for the simulations are summarized in Table S4.

We calculated multiple summary statistics for each data set using arlsumstat [35]. For the mtDNA data, we computed the number of segregating haplotypes *K*, the number of segregating sites *S*, Tajima's *D [36]*, Fu's *FS [37]*, and the average pairwise difference *π*. For the microsatellite data, we computed the total number of alleles *K*, the range of the allele size *R*, the expected heterozygosity *H* and the Garza–Williamson statistic *GW [38]*. We ensured that the observed summary statistics fell well within the distribution of simulated summary statistics (Figure S13-15).

For model-testing purposes, we performed 200,000 simulations per model using fastsimcoal2 [39]. We chose a set of informative summary statistics with a partial least-squares discriminant analysis as in [40,41] using the *plsda* function in R [42]. We compared all models (computing marginal likelihood and posterior probability) simultaneously using a standard ABC generalized linear model (GLM) approach as implemented in ABCtoolbox [43]. We also computed the average Root Mean Square Error (RMSE) for each parameter using pseudo-observed data to assess our power to infer each parameter in the model (see Table S4).

To estimate parameter values, we ran a total of 2,000,000 simulations under the best-fitting model for each species. We extracted five partial least square (PLS) components from the summary statistics in the observed and simulated data [44]. We retained a total of 10,000 simulations closest to the observed data and applied a standard ABC-GLM [45].

Supplementary Figures:

Figure S1. Venn diagram representing the number of individuals and the overlap between the various databases generated for this project. a. Anoa **b.** Babirusa **c.** SWP

Figure S2: Molecular clock results for suids alignment

Figure S3: Molecular clock results for bovids alignment

Figure S4: Bayesian phylogeny inferred from mtDNA from SWP. Support values represent posterior probabilities, S1-5 label represent haplogroups plotted in Figure 1.

Figure S5: Bayesian phylogeny based on mtDNA from Babirusa. Support values represent posterior probabilities; B1-6 labels represent haplogroups plotted in Figure 1.

Figure S6: Bayesian phylogeny based on mtDNA from Anoa. Support values represent posterior probabilities; A1-5 labels represents haplogroups plotted in Figure 1.

Figure S7: Tectonic reconstruction of Sulawesi over the last 8My in 1My increments adapted from [46]

Figure S8: ΔK **values for each species (best number of clusters in the microsatellite data). a.** Anoa **b.** Babirusa **c.** SWP.

Figure S9: Neighbour-joining trees based on Fst. a. Anoa **b.** Babirusa **c.** Sulawesi warty pig.

Figure S10: Results of the STRUCTURE analysis for K**=2 to** K**=6. a.** Anoa **b.** Babirusa **c.** Sulawesi warty pig.

Figure S11: Various models tested using approximate Bayesian computation. a. Constant population size (Model 1). **b.** Population expansion (Model 2). **c.** Population bottleneck (Model 3). **d.** Population expansion followed by a bottleneck (Model 4).

Figure S12: Neighbour-joining tree based on pairwise proportion of shared alleles using the microsatellite data. a. Anoa **b.** Babirusa **c.** SWP.

Figure S13 Observed (red vertical line) and simulated (histogram) of all summary statistics used in the approximate Bayesian computation analysis (Anoa).

Figure S14 Observed (red vertical line) and simulated (histogram) of all summary statistics used in the approximate Bayesian computation analysis (Babirusa).

Figure S15 Observed (red vertical line) and simulated (histogram) of all summary statistics used in the approximate Bayesian computation analysis (SWP).

Figure S16: Population structure of each species inferred from mtDNA, microsatellites. a. to c., Proportion of haplogroups in each region of endemism and phylogeny of Anoa (**a.**), Babirusa (**b.**) and Sulawesi warty pig (**c.**). Numbers in pie charts represent the sample size in a given region. **d. to f.,** Result of the STRUCTURE analysis using the microsatellite data plotted on the map and as a bar chart (Figure S10) for Anoa

(**d.**), Babirusa (**e.**) and SWP (**f.**). The best *K* value for each species was used (*K*=5 for Anoa; *K*=6 for Babirusa; *K*=5 for SWP). NE=North East; NC=North Central; NW=North West; TO=Togian; BA=Banggai Archipelago; EC=East Central; WC=West Central; SU=Sula; BU=Buru; S=Sula or Buru; SE=South East; SW= South West; BT=Buton.

Supplementary Tables:

Table S1: Table containing sample information for all three species – available at https://doi.org/10.5061/dryad.dv322

Table S2: Pairwise Wilcoxon tests for the lower M3 (upper part) and lower M2 (lower part), for the lower M3 (upper part) and lower M2 (lower part).

Table S3: **Support for various models obtained from the ABC analysis.** Each models tested (1-4) are displayed in Figure S11. Obs. P-value= observed fraction of the retained simulation (2,000) with a marginal likelihood value (marginal *lnL*) smaller than the observed data. Posterior *P.* = Posterior probability of the model.

Table S4: Characteristics of the prior and posterior distribution of parameters estimated via approximate Bayesian computation. All priors are uniformly distributed. The average root mean square error (RMSE) of the mode of each parameter was computed using 1,000 pseudo-observed data sets. Values close to 1 and 0 indicates little and large power, respectively. 95CI represents the 95% credibility interval. See Figure S11 for further information about the parameters.

Table S5: Results of the AMOVA based on microsatellite data.

Table S6: List of all primers used in this study

Table S7: Marginal likelihood of molecular clock analyses under constant-size coalescent prior, Bayesian skyline coalescent prior, and birth-death speciation prior.

References:

1. Groves CP. 1969 Systematics of the anoa (Mammalia, Bovidae). *Beaufortia* **17**, 1–12.

2. Cucchi T, Hulme-Beaman A, Yuan J, Dobney K. 2011 Early Neolithic pig domestication at Jiahu, Henan Province, China: clues from molar shape analyses using geometric morphometric approaches. *J. Archaeol. Sci.* **38**, 11–22.

3. Evin A, Cucchi T, Cardini A, Strand Vidarsdottir U, Larson G, Dobney K. 2013 The long and winding road: identifying pig domestication through molar size and shape. *J. Archaeol. Sci.* **40**, 735–743.

4. Cymbron T, Loftus RT, Malheiro MI, Bradley DG. 1999 Mitochondrial sequence variation suggests an African influence in Portuguese cattle. *Proc. Biol. Sci.* **266**, 597–603.

5. Schreiber A, Seibold I, Nötzold G, Wink M. 1999 Cytochrome b gene haplotypes characterize chromosomal lineages of anoa, the Sulawesi dwarf buffalo (Bovidae: Bubalus sp.). *J. Hered.* **90**, 165–176.

6. Larson G *et al.* 2005 Worldwide phylogeography of wild boar reveals multiple centers of pig domestication. *Science* **307**, 1618–1621.

7. Larson G *et al.* 2007 Ancient DNA, pig domestication, and the spread of the Neolithic into Europe. *Proc. Natl. Acad. Sci. U. S. A.* **104**, 15276–15281.

8. Bradley DG, Fries R, Bumstead N, Nicholas FW, Cothran EG, Ollivier L, Crawford AM. 2004 Secondary Guidelines for Development of National Farm Animal Genetic Resources Management Plans. *Food and Agricultural Organization of United Nations (FAO), Roma, Italy.*

9. Ronquist F *et al.* 2012 MrBayes 3.2: Efficient Bayesian Phylogenetic Inference and Model Choice Across a Large Model Space. *Syst. Biol.*, **61**, 539-542.

10. Caye K, Jay F, Michel O, Francois O. 2016 Fast Inference of Individual Admixture Coefficients Using Geographic Data. *bioRxiv* , 080291.

11. Martins H, Caye K, Luu K, Blum MGB, Francois O. 2016 Identifying outlier loci in admixed and in continuous populations using ancestral population differentiation statistics. *bioRxiv* , 054585.

12. Caye K, Deist TM, Martins H, Michel O, François O. 2016 TESS3: fast inference of spatial population structure and genome scans for selection. *Mol. Ecol. Resour.* **16**, 540–548.

13. Evans BJ, Supriatna J, Andayani N, Setiadi MI, Cannatella DC, Melnick DJ. 2003 Monkeys and toads define areas of endemism on Sulawesi. *Evolution* **57**, 1436–1443.

14. Evans BJ, Supriatna J, Andayani N, Melnick DJ. 2003 Diversification of Sulawesi macaque monkeys: decoupled evolution of mitochondrial and autosomal DNA. *Evolution* **57**, 1931–1946.

15. Merker S, Driller C, Perwitasari-Farajallah D, Pamungkas J, Zischler H. 2009 Elucidating geological and biological processes underlying the diversification of Sulawesi tarsiers. *Proc. Natl. Acad. Sci. U. S. A.* **106**, 8459–8464.

16. Drummond AJ, Suchard MA, Xie D, Rambaut A. 2012 Bayesian Phylogenetics with BEAUti and the BEAST 1.7. *Mol. Biol. Evol.* **29**, 1969–1973.

17. Gongora J *et al.* 2011 Rethinking the evolution of extant sub-Saharan African suids (Suidae, Artiodactyla). *Zool. Scr.* **40**, 327–335.

18. Bibi F. 2013 A multi-calibrated mitochondrial phylogeny of extant Bovidae (Artiodactyla, Ruminantia) and the importance of the fossil record to systematics. *BMC Evol. Biol.* **13**, 166.

19. Ho SYW, Lanfear R, Bromham L, Phillips MJ, Soubrier J, Rodrigo AG, Cooper A. 2011 Time-dependent rates of molecular evolution. *Mol. Ecol.* **20**, 3087–3101.

20. Drummond AJ, Ho SYW, Phillips MJ, Rambaut A. 2006 Relaxed phylogenetics and dating with confidence. *PLoS Biol.* **4**, e88.

21. Baele G, Lemey P, Bedford T, Rambaut A, Suchard MA, Alekseyenko AV. 2012 Improving the Accuracy of Demographic and Molecular Clock Model Comparison While Accommodating Phylogenetic Uncertainty. *Mol. Biol. Evol.* **29**, 2157–2167.

22. Pritchard JK, Stephens M, Donnelly P. 2000 Inference of population structure using multilocus genotype data. *Genetics* **155**, 945–959.

23. Earl DA, vonHoldt BM. 2011 STRUCTURE HARVESTER: a website and program for visualizing STRUCTURE output and implementing the Evanno method. *Conserv. Genet. Resour.* **4**, 359–361.

24. Jakobsson M, Rosenberg NA. 2007 CLUMPP: a cluster matching and permutation program for dealing with label switching and multimodality in analysis of population structure. *Bioinformatics* **23**, 1801–1806.

25. Goudet J. 2005 Hierfstat, a package for R to compute and test hierarchical F-statistics. *Mol. Ecol. Resour.* **5**, 184–186.

26. Weir BS, Cockerham CC. 1984 Estimating F-Statistics for the Analysis of Population Structure. *Evolution* **38**, 1358.

27. Excoffier L, Smouse PE, Quattro JM. 1992 Analysis of molecular variance inferred from metric distances among DNA haplotypes: application to human mitochondrial DNA restriction data. *Genetics* **131**.

28. Kamvar ZN, Tabima JF, Grünwald NJ. 2014 *Poppr* : an R package for genetic analysis of populations with clonal, partially clonal, and/or sexual reproduction. *PeerJ* **2**, e281.

29. Dray S, Dufour A-B. 2007 The **ade4** Package: Implementing the Duality Diagram for Ecologists. *J. Stat. Softw.* **22**, 1–20.

30. Bowcock AM, Ruiz-Linares A, Tomfohrde J, Minch E, Kidd JR, Cavalli-Sforza LL. 1994 High resolution of human evolutionary trees with polymorphic microsatellites. *Nature* **368**, 455–457.

31. Felsenstein J. 1989 PHYLIP - Phylogeny Inference Package (Version 3.2). *Cladistics* **5**, 163–166.

32. Goldstein DB, Ruiz Linares A, Cavalli-Sforza LL, Feldman MW. 1995 An evaluation of genetic distances for use with microsatellite loci. *Genetics* **139**, 463–471.

33. Sun JX, Mullikin JC, Patterson N, Reich DE. 2009 Microsatellites are molecular clocks that support accurate inferences about history. *Mol. Biol. Evol.* **26**, 1017–1027.

34. Dieringer D, Schlötterer C. 2003 microsatellite analyser (MSA): a platform independent analysis tool for large microsatellite data sets. *Mol. Ecol. Notes* **3**, 167–169.

35. Excoffier L, Lischer HEL. 2010 Arlequin suite ver 3.5: a new series of programs to perform population genetics analyses under Linux and Windows. *Mol. Ecol. Resour.* **10**, 564–567.

36. Tajima F. 1989 Statistical method for testing the neutral mutation hypothesis by DNA polymorphism. *Genetics* **123**, 585–595.

37. Fu YX. 1997 Statistical Tests of Neutrality of Mutations Against Population Growth, Hitchhiking and Background Selection. *Genetics* **147**, 915–925.

38. Garza JC, Williamson EG. 2001 Detection of reduction in population size using data from microsatellite loci. *Mol. Ecol.* **10**, 305–318.

39. Excoffier L, Foll M. 2011 fastsimcoal: a continuous-time coalescent simulator of genomic diversity under arbitrarily complex evolutionary scenarios. *Bioinformatics* **27**, 1332–1334.

40. Peter BM, Huerta-Sanchez E, Nielsen R. 2012 Distinguishing between selective sweeps from standing variation and from a de novo mutation. *PLoS Genet.* **8**, e1003011.

41. Frantz LAF *et al.* 2015 Evidence of long-term gene flow and selection during domestication from analyses of Eurasian wild and domestic pig genomes. *Nat. Genet.* **47**, 1141–1148.

42. Lê Cao K-A, González I, Déjean S. 2009 integrOmics: an R package to unravel relationships between two omics datasets. *Bioinformatics* **25**, 2855–2856.

43. Wegmann D, Leuenberger C, Neuenschwander S, Excoffier L. 2010 ABCtoolbox: a versatile toolkit for approximate Bayesian computations. *BMC Bioinformatics* **11**, 116.

44. Wegmann D, Leuenberger C, Excoffier L. 2009 Efficient approximate Bayesian computation coupled with Markov chain Monte Carlo without likelihood. *Genetics* **182**, 1207–1218.

45. Leuenberger C, Wegmann D. 2010 Bayesian computation and model selection without likelihoods. *Genetics* **184**, 243–252.

46. Abang Mansyursyah Surya Nugraha and Robert Hall. In press. Late Cenozoic palaeogeography of Sulawesi, Indonesia. *Palaeogeogr. Palaeoclimatol. Palaeoecol.*

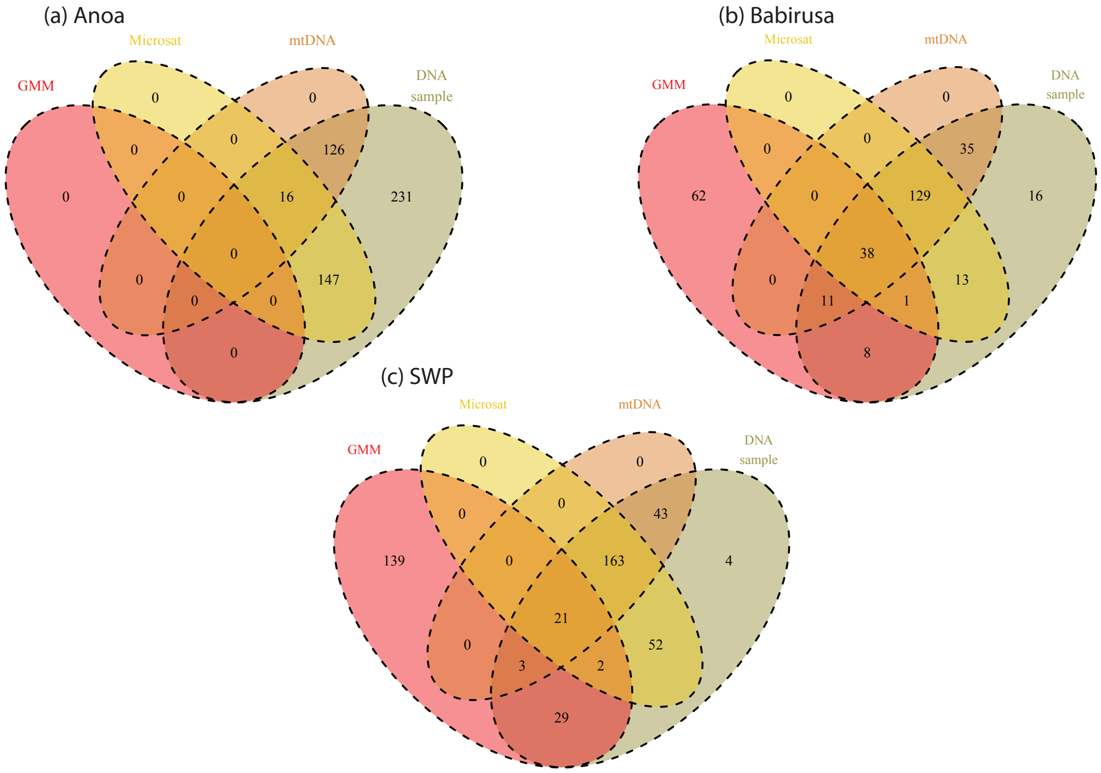

Figure S1. Venn diagram representing the number of individuals and the overlap between the various databases generated for this project. a. Anoa **b.** Babirusa **c.** SWP.

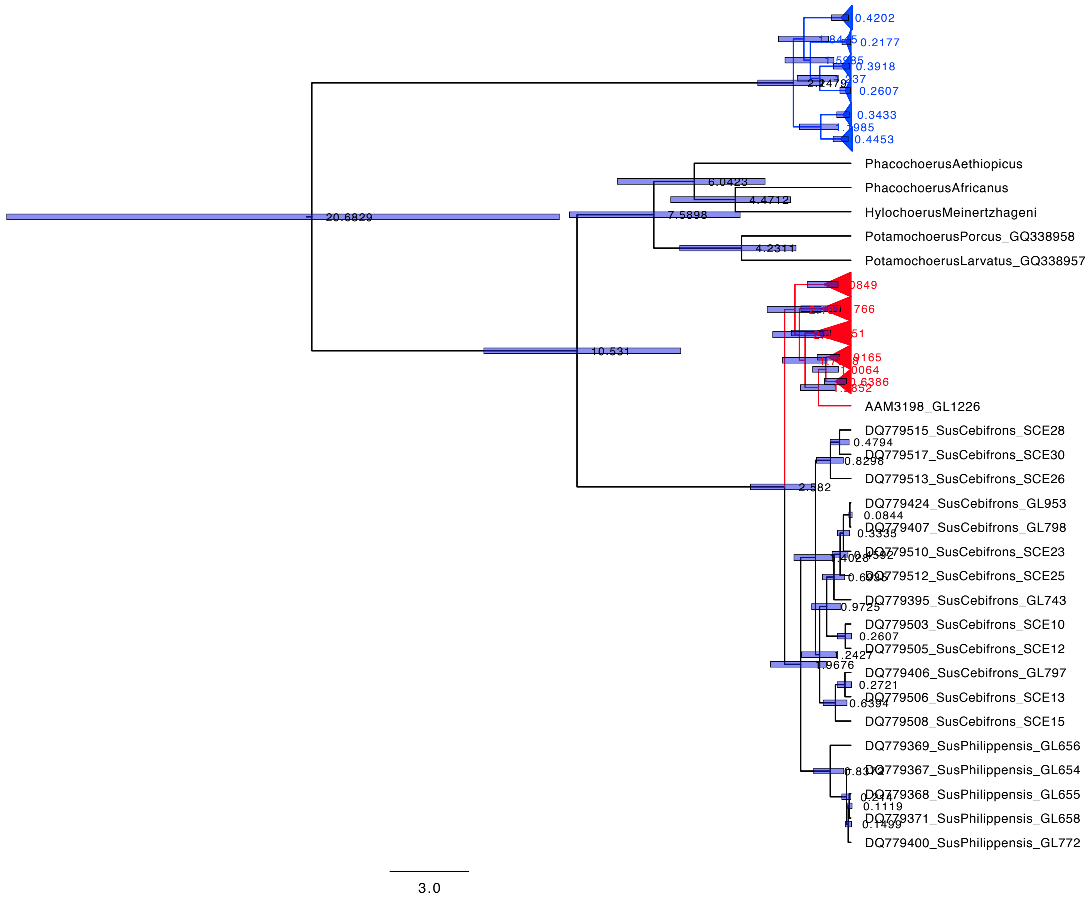

Figure S2: Molecular clock results for suids alignment

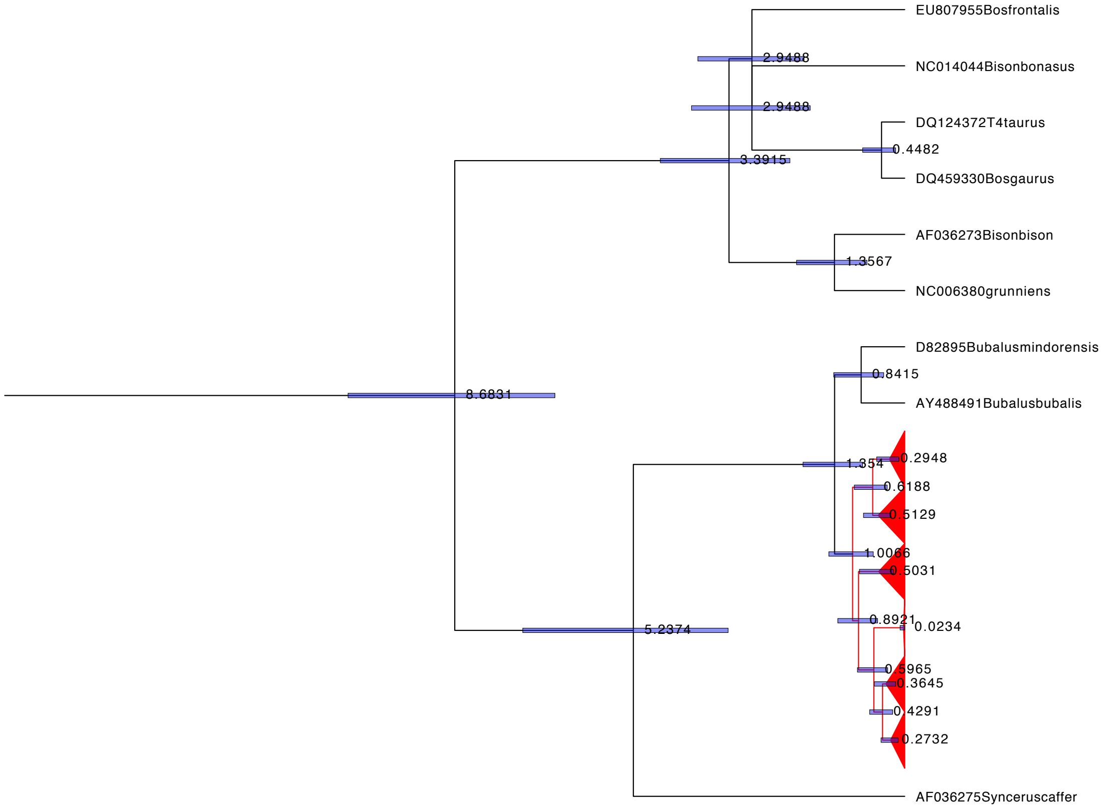

Figure S3: Molecular clock results for bovids alignment

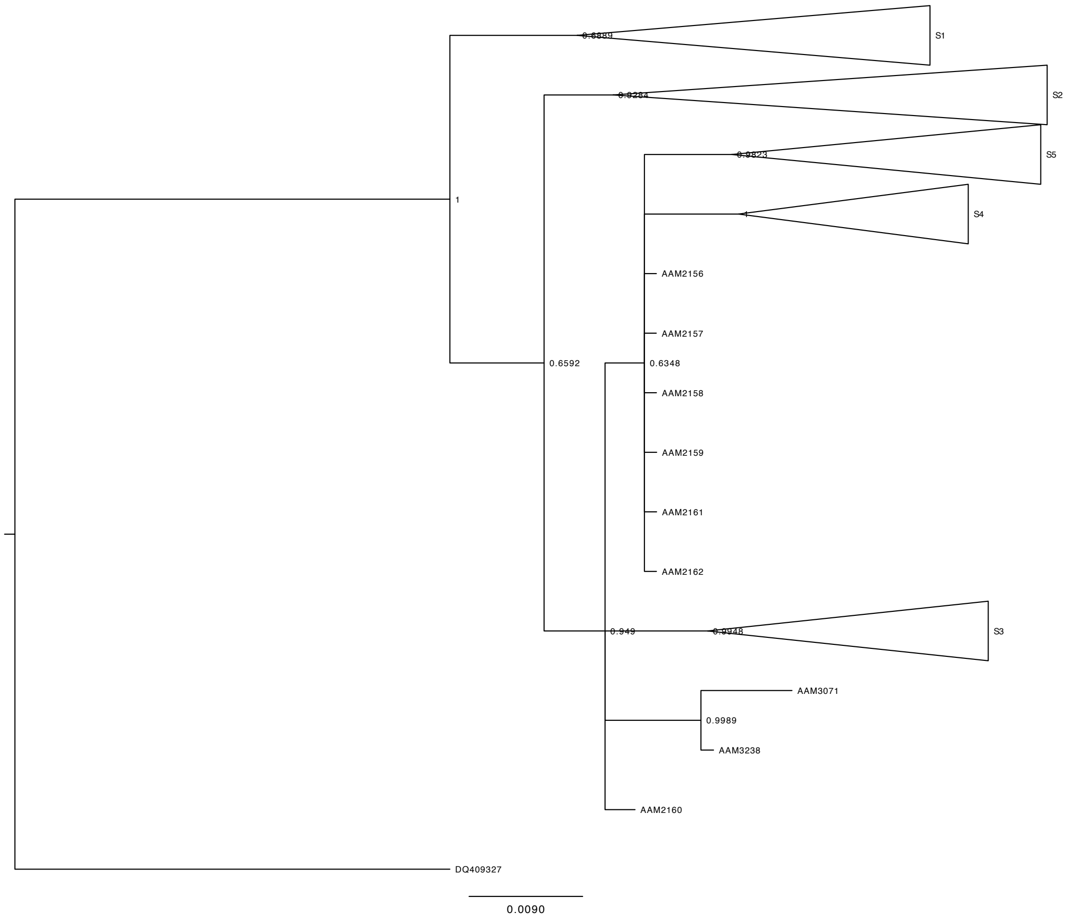

Figure S4: Bayesian phylogeny inferred from mtDNA from SWP. Support values represent posterior probabilities, S1-5 label represent haplogroups plotted in Fig. 1.

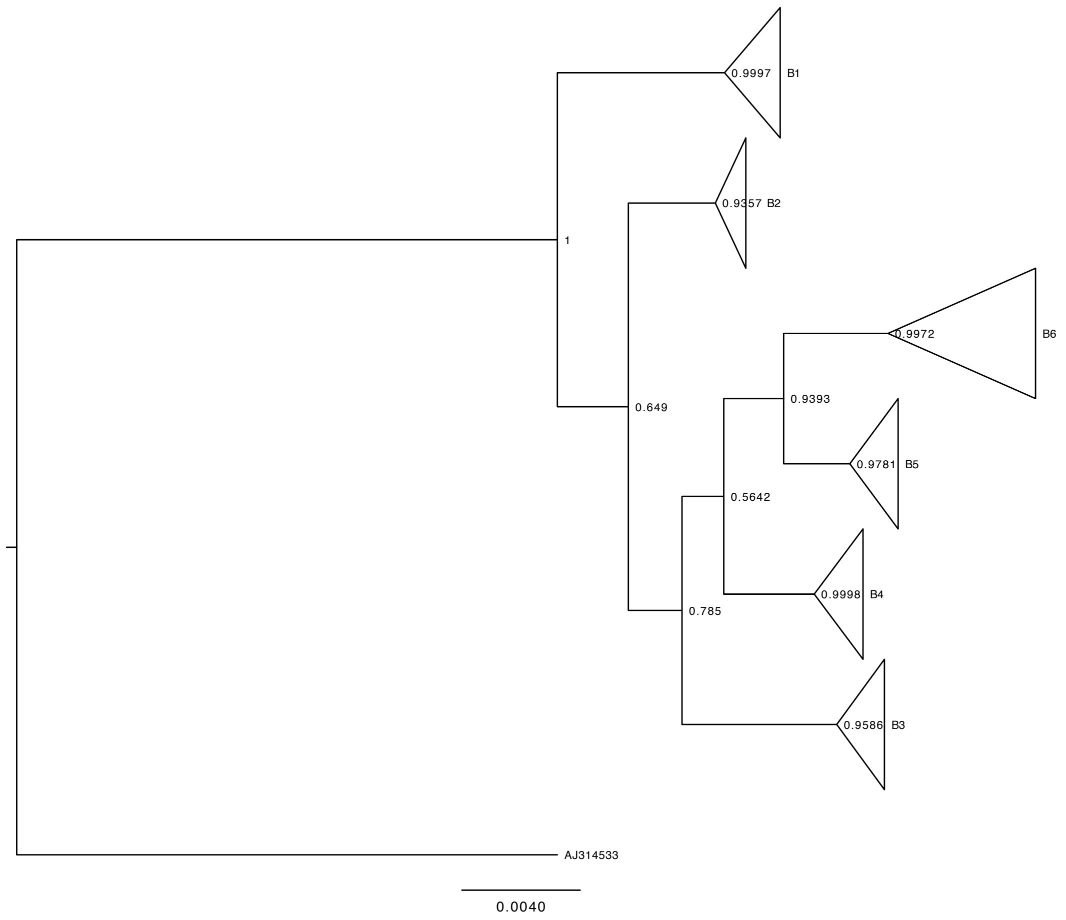

Figure S5: Bayesian phylogeny based on mtDNA from Babirusa. Support values represent posterior probabilities; B1-6 labels represent haplogroups plotted in Fig. 1.

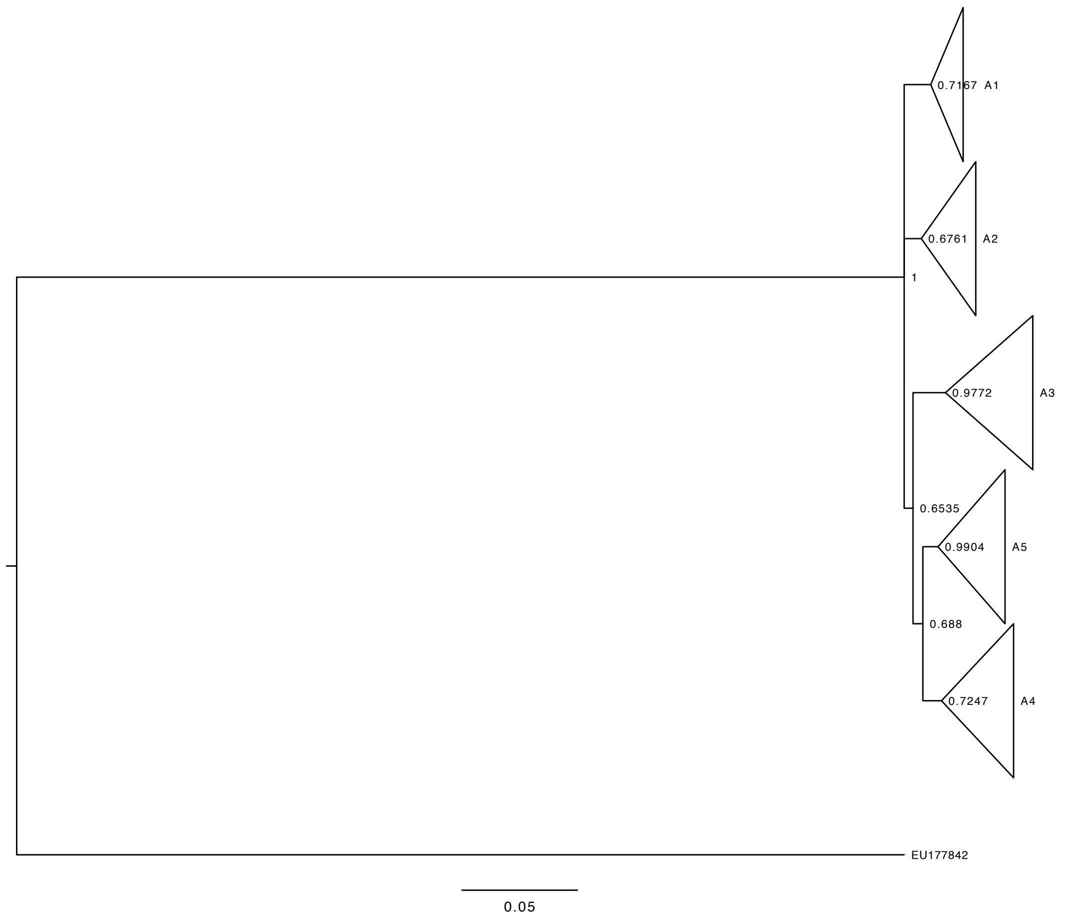

Figure S6: Bayesian phylogeny based on mtDNA from Anoa. Support values represent posterior probabilities; A1-5 labels represents haplogroups plotted in Fig. 1.

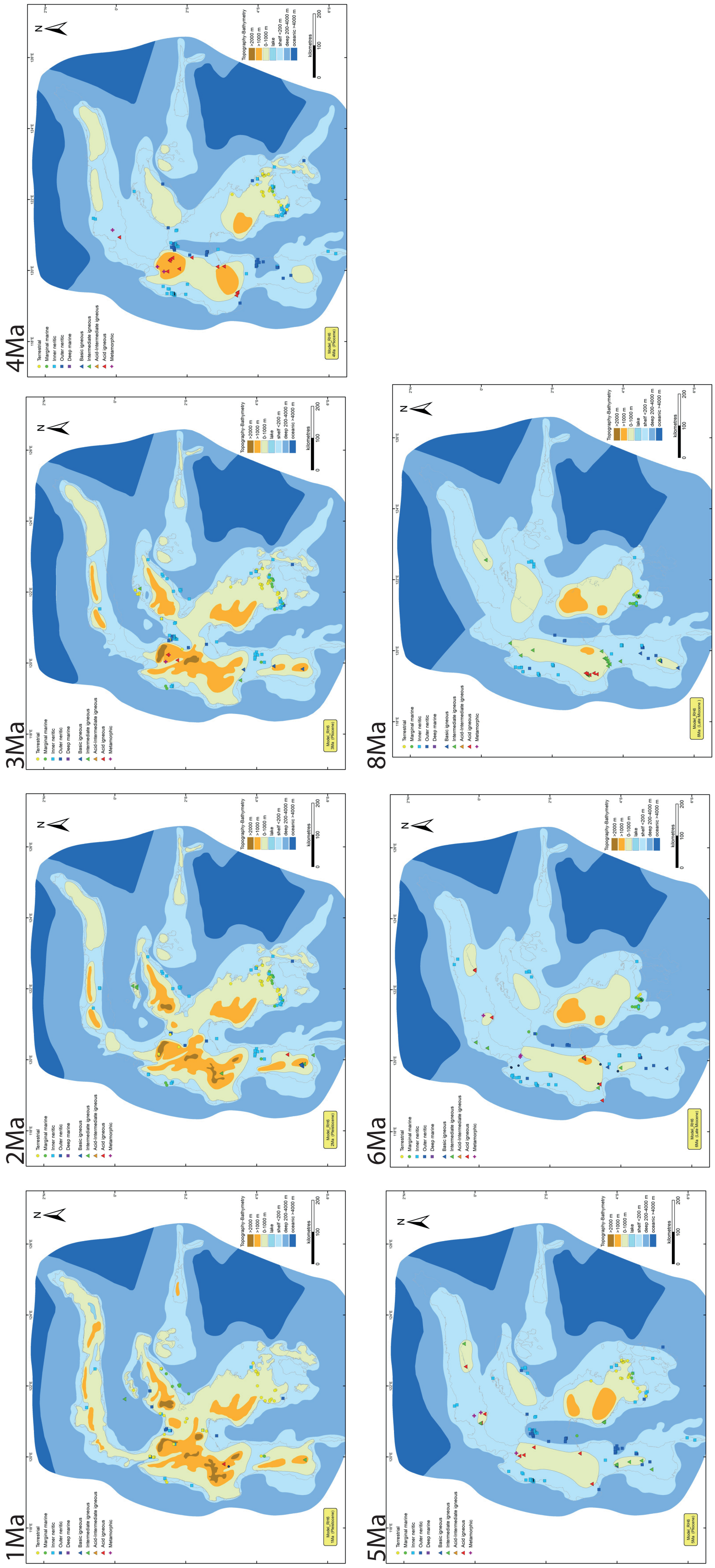

Figure S7: Tectonic reconstruction of Sulawesi over the last 8My in 1My increments.

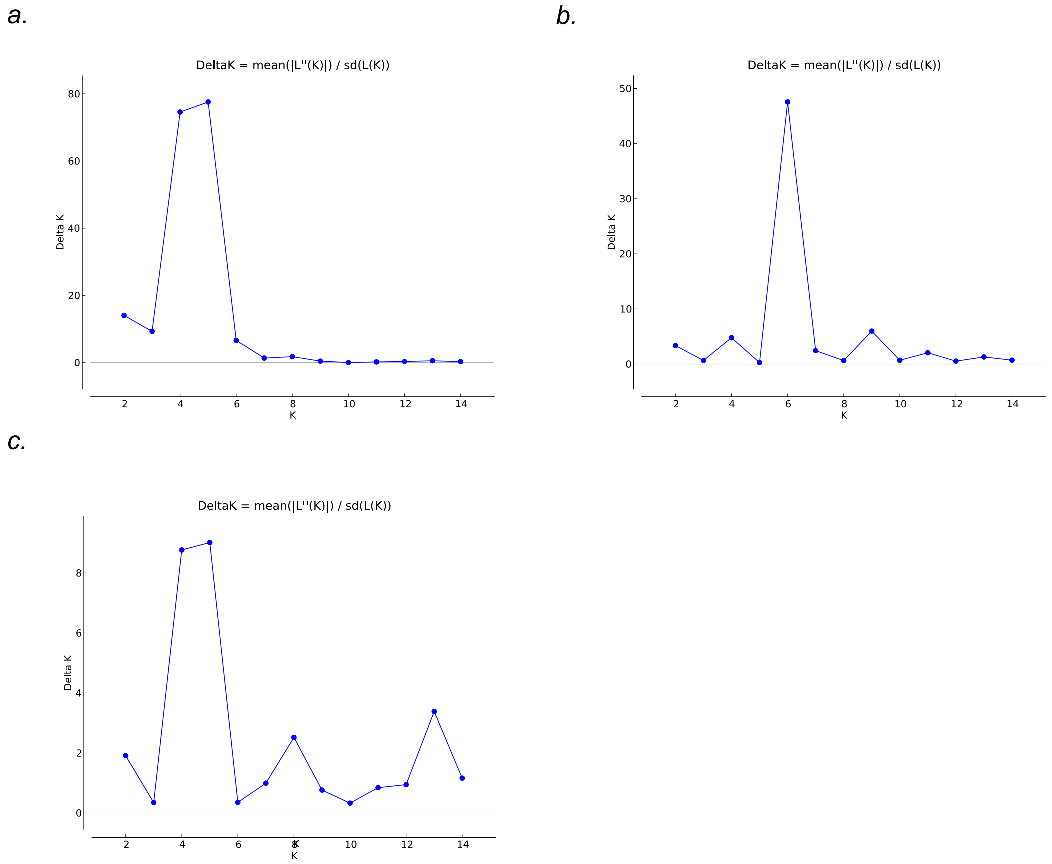

Figure S8: Δ*K* values for each species (best number of clusters in the microsatellite data). a. Anoa **b.** Babirusa **c.** Sulawesi warty pig.

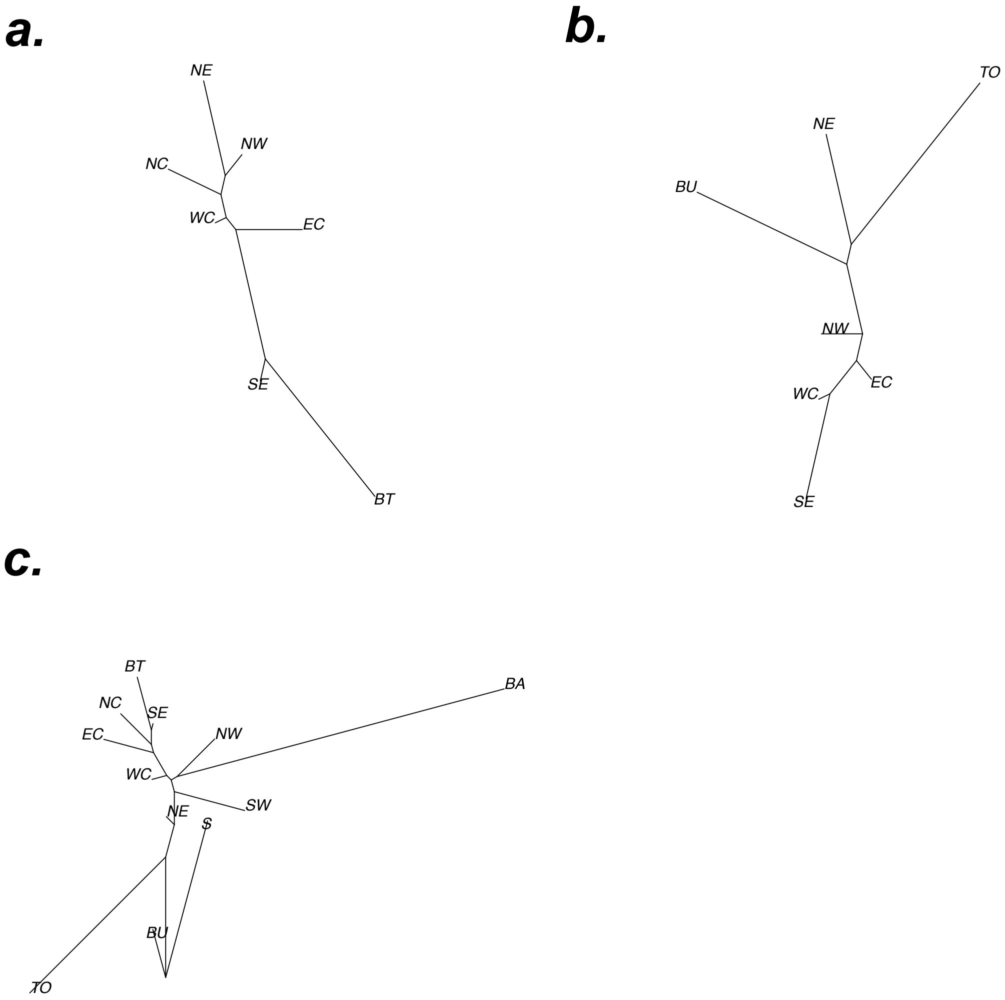

Figure S9: Neighbour-joining trees based on Fst. a. Anoa **b.** Babirusa **c.** Sulawesi warty pig.

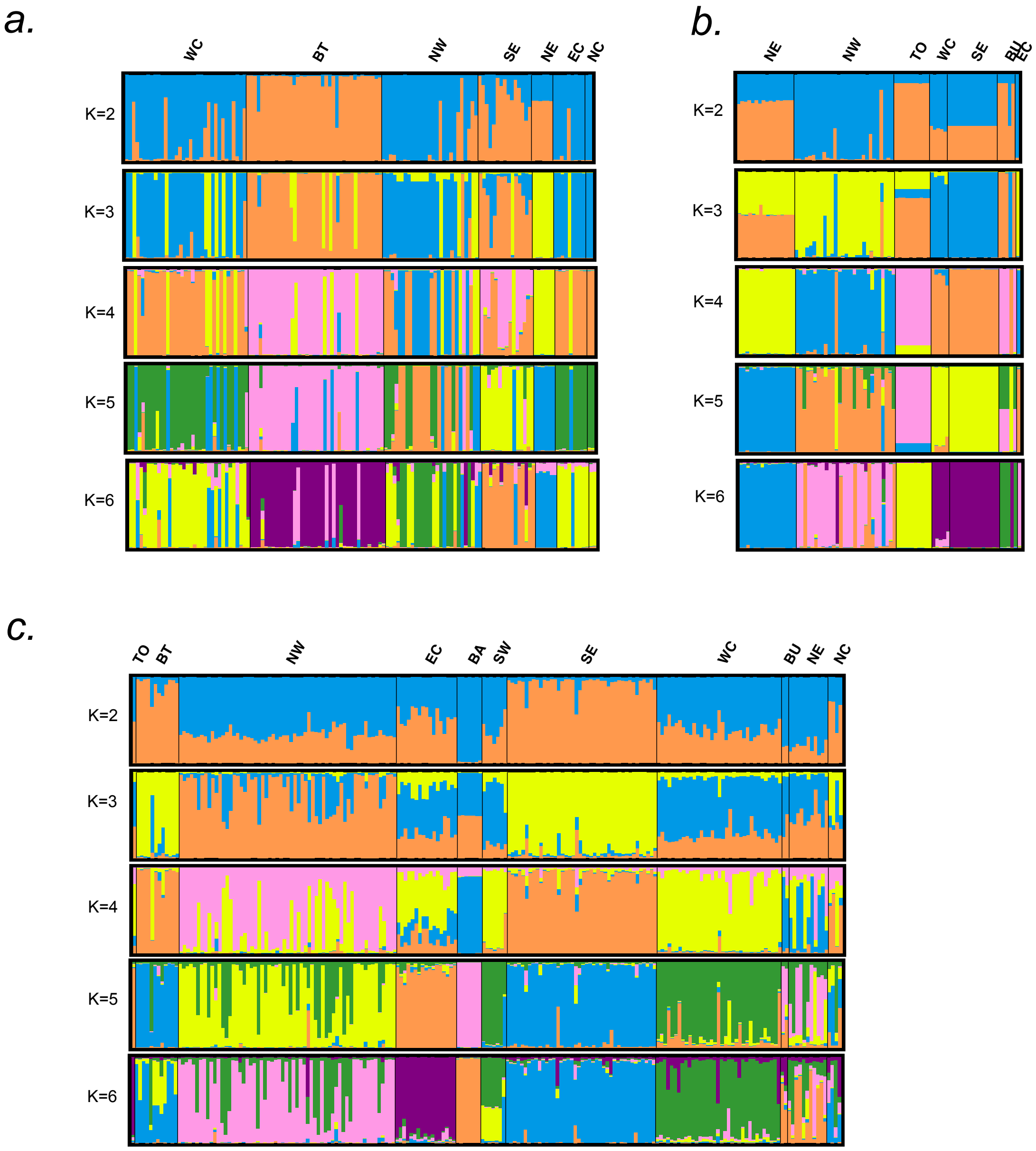

Figure S10: Results of the STRUCTURE analysis for *K*=2 to *K*=6. a. Anoa **b.** Babirusa **c.** SWP*.*

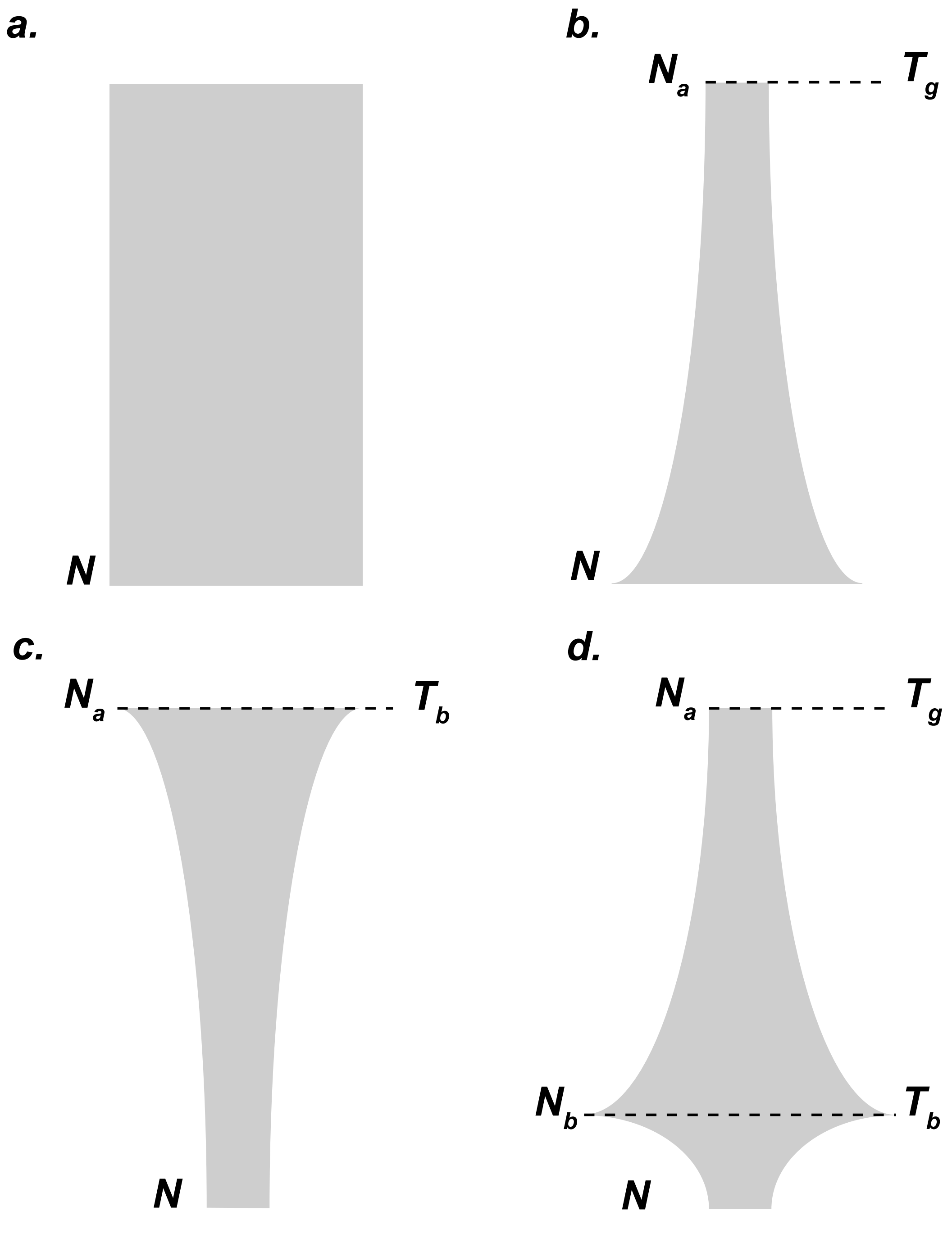

Figure S11: Various models tested using approximate Bayesian computation. a. Constant population size (Model 1). **b.** Population expansion (Model 2). **c.** Population bottleneck (Model 3). **d.** Population expansion followed by a bottleneck (Model 4).

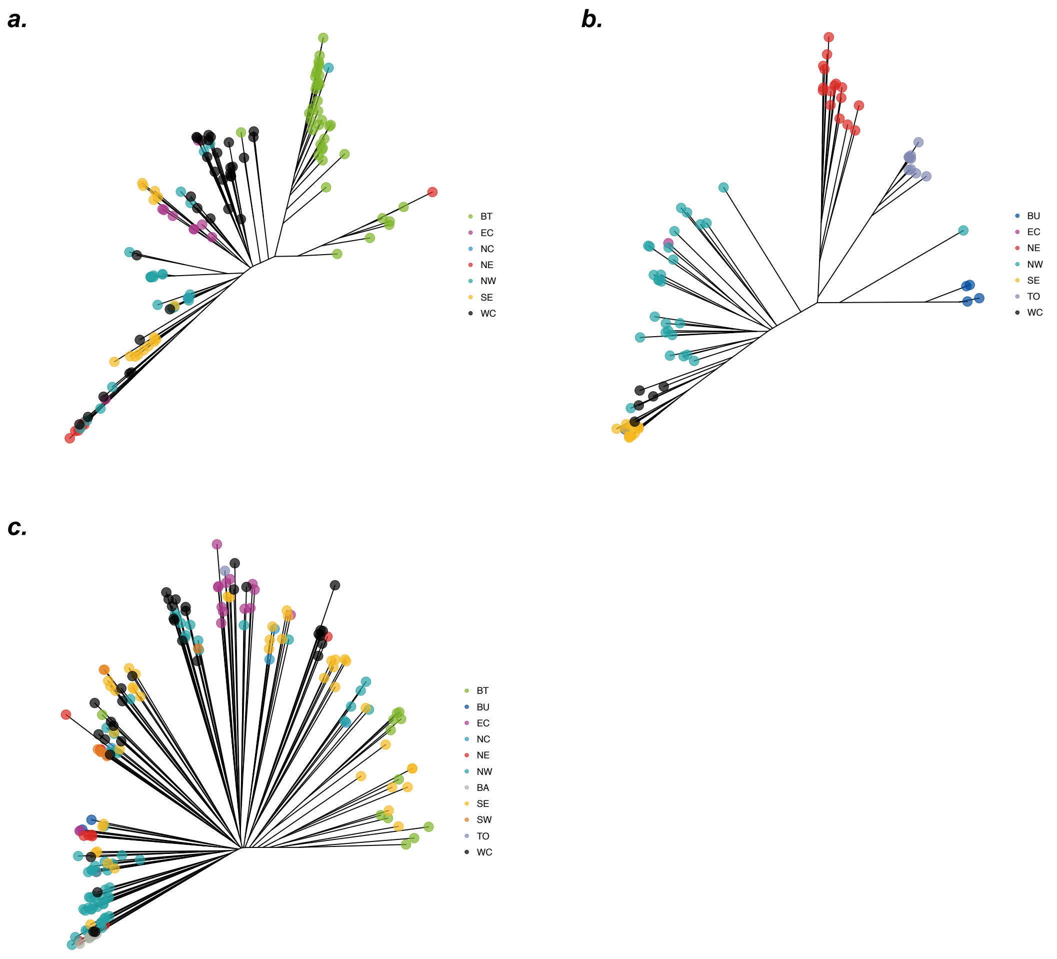

Figure S12: Neighbour-joining tree based on pairwise proportion of shared alleles using the microsatellite data. a. Anoa **b.** Babirusa **c.** Sulawesi warty pig.

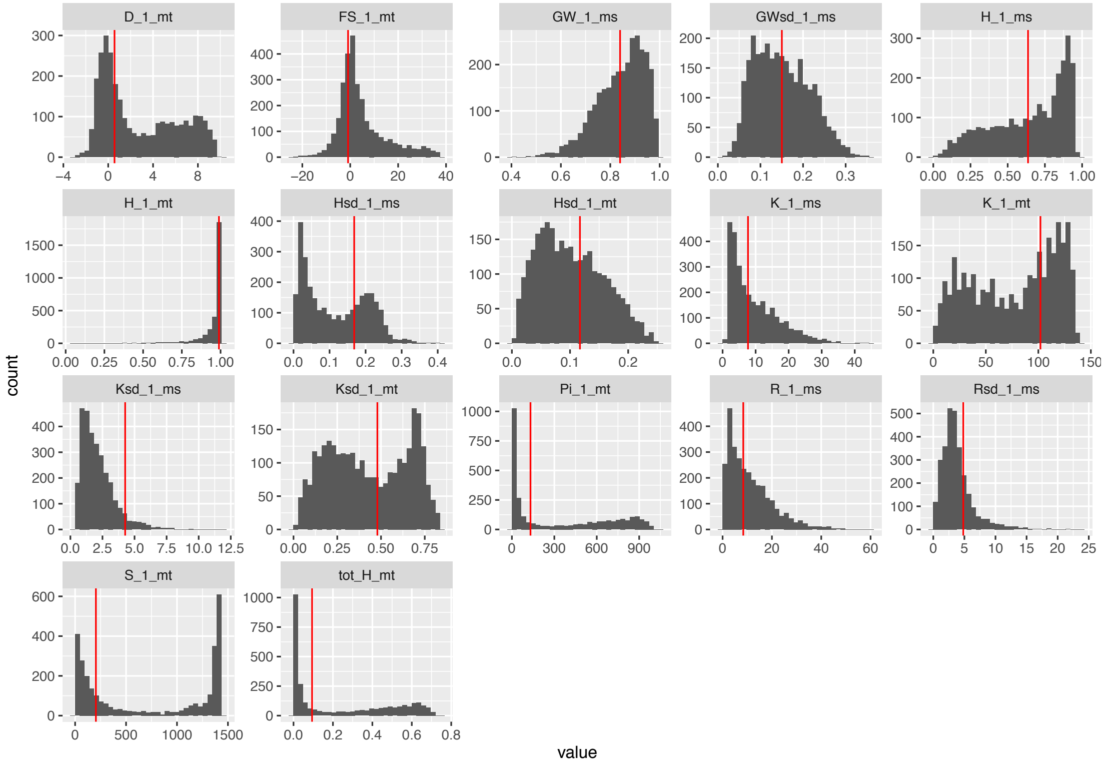

Figure S13 Observed (red vertical line) and simulated (histogram) of all summary statistics used in the approximate Bayesian computation analysis (Anoa).

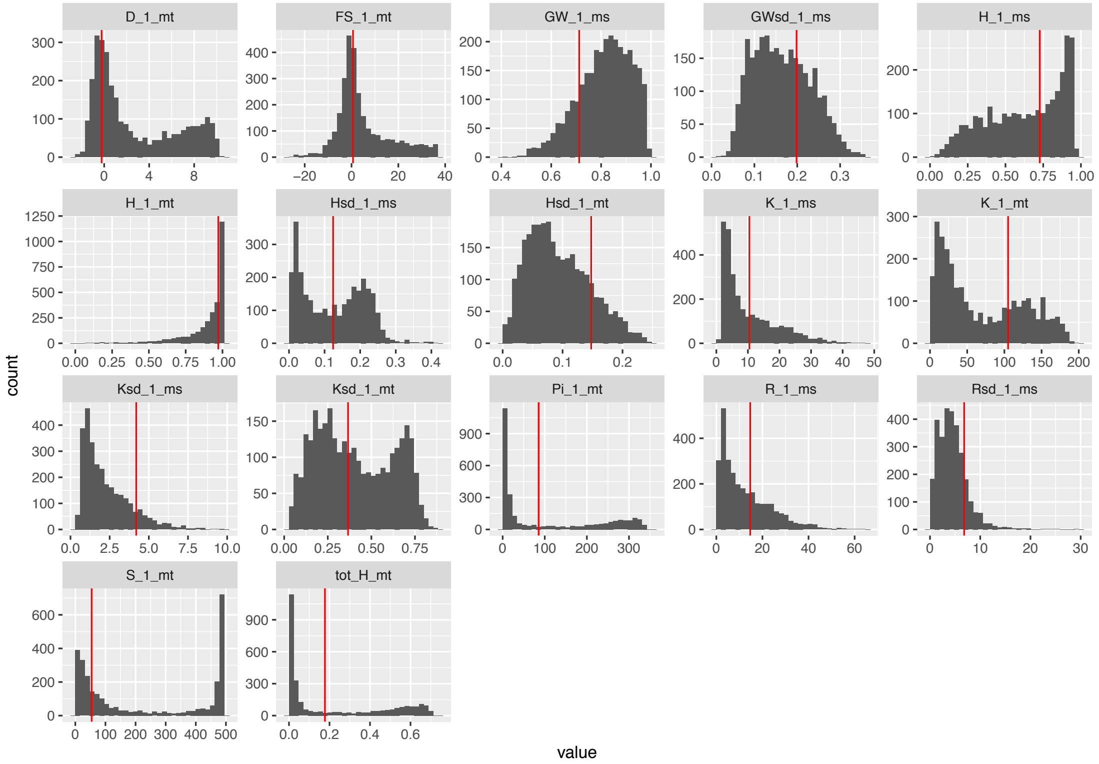

Figure S14 Observed (red vertical line) and simulated (histogram) of all summary statistics used in the approximate Bayesian computation analysis (Babirusa).

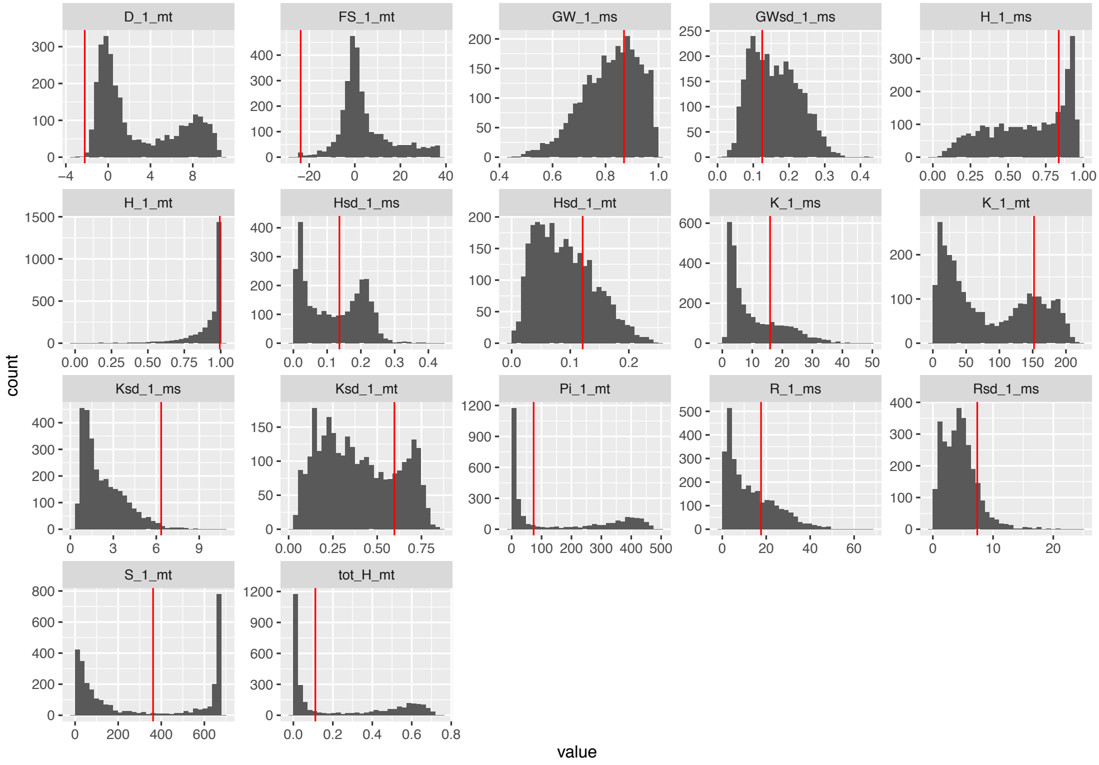

Figure S15 Observed (red vertical line) and simulated (histogram) of all summary statistics used in the approximate Bayesian computation analysis (SWP).

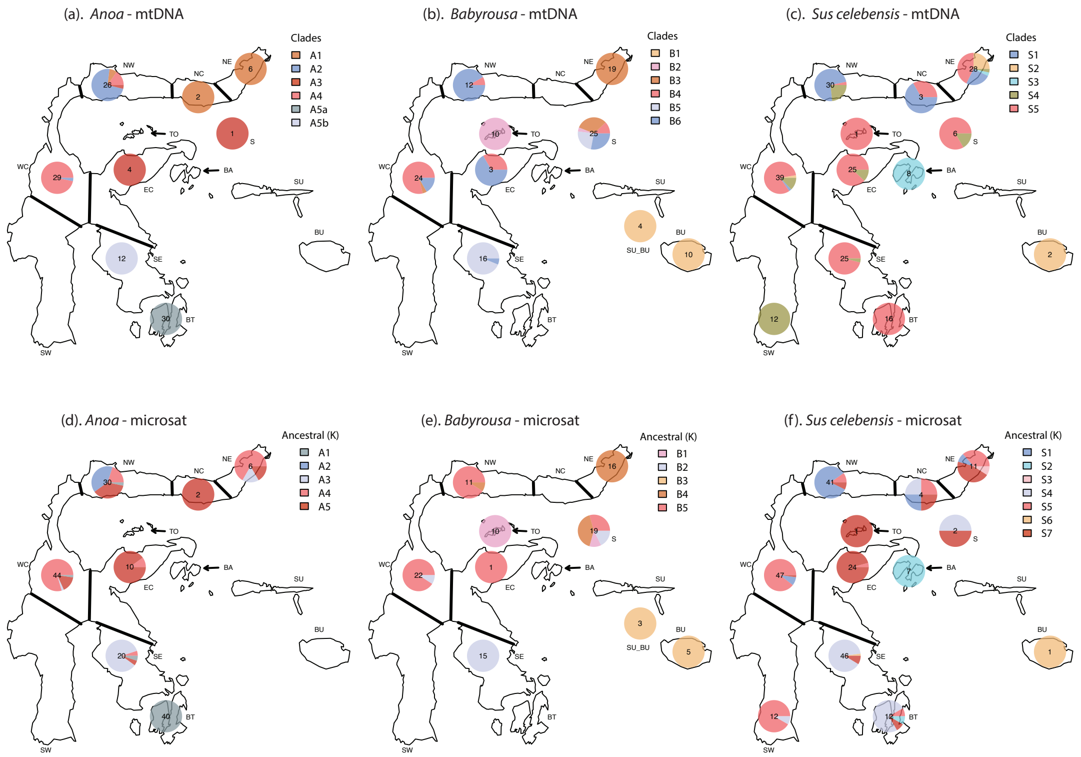

Figure S16: Population structure of each species inferred from mtDNA, microsatellites. a. to c., Proportion of haplogroups in each region of endemism and phylogeny of Anoa (**a.**), Babirusa (**b.**) and Sulawesi warty pig (**c.**). Numbers in pie charts represent the sample size in a given region. **d. to f.,** Result of the STRUCture analysis using the microsatellite data plotted on the map and as a bar chart (Fig. S10) for Anoa (**d.**), Babirusa (**e.**) and SWP (**f.**). The best *K* value for each species was used (*K*=5 for Anoa; *K*=6 for Babirusa; *K*=5 for SWP). NE=North East; NC=North Central; NW=North West; TO=Togian; BA=Banggai Archipelago; EC=East Central; WC=West Central; SU=Sula; BU=Buru; S=Sula or Buru; SE=South East; SW= South West; BT=Buton.

Table S2: Pairwise Wilcoxon tests for the lower M3 (upper part) and lower M2 (lower part), for the lower M3 (upper part) and lower M2 (lower part).

	Bab.West_Central	Bab.North_West	Bab.North_East	Bab.Sula_Buru	Bab.Togian	Sus.West_Central	Sus.North_West	Sus.North_East	Sus.Banggai
Bab.West_Centra	-	0.950	0.428	0.622	0.950	0.499	0.130	0.347	0.435
Bab.North_West	0.950	-	0.664	0.429	0.699	0.132	0.142	0.420	0.429
Bab.North_East	0.332	0.634	-	0.202	0.520	0.004	0.104	0.633	0.598
Bab.Sula_Buru	0.354	0.247	0.048	-	0.931	0.511	0.019	0.206	0.151
Bab.Togian	0.001	0.004	0.000	0.052	-	0.098	0.059	0.420	0.247
Sus.West_Centra	0.001	0.003	0.000	0.087	0.508	-	0.003	0.006	0.046
Sus.North_West	0.798	0.852	1.000	0.435	0.020	0.007	-	0.261	0.435
Sus.North_East	0.224	0.451	0.363	0.026	0.000	0.000	0.491	-	0.931
Sus.Banggai	0.524	0.662	0.105	0.841	0.017	0.077	0.354	0.068	-

Table S3: Support for various models obtained from the ABC analysis.

		Obs. *P*-value	Marginal *lnL*	Bayes Factor	Posterior *P.*
Bubalus spp.	Model 1	0	6.78E-08	4.04E-05	4.04E-05
	Model 2	0	1.00E-08	5.97E-06	5.97E-06
	Model 3	0.379	0.000365477	0.278348	0.21774
	Model 4	0.8	0.00131294	**3.59165**	**0.782213**
Babyroussa spp.	Model 1	0	8.89E-16	8.43E-13	8.43E-13
	Model 2	0	2.40E-16	2.28E-13	2.28E-13
	Model 3	0.406	0.00033359	0.462939	0.316444
	Model 4	0.673	0.000720592	**2.16011**	**0.683556**
S. celebensis	Model 1	0	3.01E-09	3.87E-05	3.87E-05
	Model 2	0	4.78E-09	6.15E-05	6.15E-05
	Model 3	0.026	1.25E-05	0.190926	0.160317
	Model 4	0.087	6.53E-05	**5.23374**	**0.839583**

Table S4: Characteristics of the prior and posterior distribution of parameters estimated via approximate Bayesian computation.

	parameter	prior_min	prior_max	RMSE	mode	HPDI-95- lower	HPDI-95- upper
B. depressicornis	N	3	5.5	0.3455	4.4394	4.13611	4.73285
	N_a/N_b	-3	0	0.9441	-1.39394	-2.98221	-0.160913
	N_b/N	0	2	0.9488	1.23232	1.02401	1.93015
	T_g	130000	440000	0.9791	233334	140986	424006
	T_b	1	15000	0.887	11970	2896	14671
B. babirussa	N	3	5.5	0.3234	4.4899	4.2436	4.74727
	N_a/N_b	-3	0	0.9774	-1.87879	-2.89784	-0.17991
	N_b/N	0	2	0.9084	1.29293	1.03074	1.93997
	T_g	330000	940000	0.9909	570303	352200	910694
	T_b	1	15000	0.8978	13485	5370	14832
S. celebensis	N	3	5.5	0.3098	4.91919	4.66545	5.2083
	N_a/N_b	-3	0	0.9795	-2.06061	-2.90735	-0.188233
	N_b/N	0	2	0.9171	1.23232	1.02349	1.92281
	T_g	330000	940000	0.995	521010	349250	904971
	T_b	1	15000	0.8942	11212	3016	14597

Table S5: Results of the AMOVA based on microsatellite data.

AMOVA Bubalus spp.

	Sigma	%
Variations Between Population	0.40	17.31
Variations Between samples Within Population	0.59	25.44
Variations Within samples	1.32	57.26
Total variations	2.31	100.00

AMOVA Babyroussa spp.

	Sigma	%
Variations Between Population	1.04	27.70
Variations Between samples Within Population	0.13	3.34
Variations Within samples	2.60	68.96
Total variations	3.77	100.00

AMOVA *S. celebensis*

	Sigma	%
Variations Between Population	0.19	4.88
Variations Between samples Within Population	0.48	12.33
Variations Within samples	3.24	82.79
Total variations	3.92	100.00

Table S6: Primers for each species

Anoa Microsatellite

Locus	Forward primer	Reverse Primer
TGLA227	CGAATTCCAAATCTGTTAATTTGCT	ACAGACAGAAACTCAATGAAAGCA
CSRM60	AAGATGTGATCCAAGAGAGAGGCA	AGGACCAGATCGTGAAAGGCATAG
TGLA126	CTAATTTAGAATGAGAGAGGCTTCT	TTGGTCTCTATTCTCTGAATATTCC
INRA037	GATCCTGCTTATATTTAACCAC	AAAATTCCATGGAGAGAGAAAC
INRA035	ATCCTTTGCAGCCTCCACATTG	TTGTGCTTTATGACACTATCCG
HEL13	AAGGACTTGAGATAAGGAG	CCATCTACCTCCATCTTAAC
MM 12	CAAGACAGGTGTTTCAATCT	ATCGACTCTGGGGATGATGT
HAUT24	CTCTCTGCCTTTGTCCCTGT	AATACACTTTAGGAGAAAAATA
HAUT27	TTTTATGTTCATTTTTTGACTGG	AACTGCTGAAATCTCCATCTTA
ILSTS5	GGAAGCAATGAAATCTATAGCC	TGTTCTGTGAGTTTGTAAAGC
ETH 152	AGGGAGGGTCACCTCTGC	CTTGTACTCGTAGGGCAGGC
SPS 115	AAAGTGACACAACAGCTTCTCCAG	AACGAGTGTCCTAGTTTGGCTGTG
BM1818	AGCTGGGAATATAACCAAAGG	AGTGCTTTCAAGGTCCATGC

Sus/Babyrousa Microsatellite

Locus	Forward primer	Reverse Primer
S0386	TCCTGGGTCTTATTTTCTA	TTTTTATCTCCAACAGTAT
S0155	TGTTCTCTGTTTCTCCTCTGTTTG	AAAGTGGAAAGAGTCAATGGCTAT
SW911	CTCAGTTCTTTGGGACTGAACC	CATCTGTGGAAAAAAAAAGCC
S0215	TAGGCTCAGACCCTGCTGCAT	TGGGAGGCTGAAGGATTGGGT
S0214	CCCTGCAAGCGTTCATCTCA	CCCTGCAAGCGTTCATCTCA
S0026	AACCTTCCCTTCCCAATCAC	CACAGACTGCTTTTTACTCC
S0149	ATTGGCTCATGAACCACCATC	GAGTTACTAATTGCCTCAGAG
S0228	GGCATAGGCTGGCAGCAACA	AGCCCACCTCATCTTATCTACACT
SW72	ATCAGAACAGTGCGCCGT	TTTGAAAATGGGGTGTTTCC
SW632	TGGGTTGAAAGATTTCCCAA	GGAGTCAGTACTTTGGCTTGA
SW951	TTTCACAACTCTGGCACCAG	GATCGTGCCCAAATGGAC
SW857	TGAGAGGTCAGTTACAGAAGACC	GATCCTCCTCCAAATCCCAT
SW936	TCTGGAGCTAGCATAAGTGCC	GTGCAAGTACACATGCAGGG
SW240	AGAAATTAGTGCCTCAAATTGG	AAACCATTAAGTCCCTAGCAAA

Anoa mtDNA

**Locus**	**Name**	**Sequence**	**F/R**	**Reference**
d-loop	AN4	GGTAATGTACATAACATTAATG	F	Cymbron 1999
d-loop	AN3	CGAGATGTCTTATTTAAGAGG	R	Cymbron 1999
d-loop	BethBigF-ww	ACMCCCAAAGCTGAAGTTCT	F	This study
d-loop	A-DL-R2c	GGTTGCTGGTTTCACGCGG	R	This study
Cyt-B	mta	CTCCCAGCCCCATCCAACATCTCAGCATGATGAAACTTCG	F	Schreiber 1999
Cyt-B	mtb	TTGTGATTACTGTAGCACCTCAAAATGATATTTGTCCTCA	R	Schreiber 1999
Cyt-B	A-CB-F2a	GCCACAGCATTTATAGGATACG	F	This study
Cyt-B	A-CB-R2a	GATCGTARGATTGCGTATGC	R	This study

Sus/Babyrousa mtDNA

S. celebensis

**Locus**	**Name**	Sequence	**F/R**	**Reference**
d-loop	L15387	CTCCGCCATCAGCACCCAAAG	F	Larson 2005
d-loop	H764	TGCTGGTTTCACGCGGCA	R	Larson 2005
d-loop	L119n	ATTATTRATCGTACATAGCAC	F	Larson 2007
d-loop	H16108n	GCACCTTGTTTGGATTRTCG	R	Larson 2007

Babirussa

d-loop	L15387	CTCCGCCATCAGCACCCAAAG	F	Larson 2005
d-loop	H648n	GCTYATATGCATGGGGACT	R	Larson 2007
d-loop	BabyF	TGTACGCCAAAACATCAAGTAC	F	This study
d-loop	RuminR	GGGCGATTTTAGGTGAGATGG	R	This study

Table S7: Marginal likelihood of molecular clock analyses under different models

Clock model	Tree prior	Marginal likelihood for bovid data set	Marginal likelihood for suid data set
Strict	Constant size coalescent	-3283.86	-5861.07
Strict	Skyline coalescent	**-3261.51**	**-5847.15**
Strict	Birth-death process	-3277.08	-5857.65
Relaxed	Constant size coalescent	-3281.97	-5856.66
Relaxed	Skyline coalescent	-3261.53	-5851.94
Relaxed	Birth-death process	-3280.03	-5863.33
